# Probable chronic renal failure caused by *Lonomia* caterpillar envenomation

**DOI:** 10.1186/1678-9199-19-14

**Published:** 2013-06-03

**Authors:** Poliana Abrantes Schmitberger, Tássia Clara Fernandes, Robson Corrêa Santos, Rafael Campos de Assis, Andréia Patrícia Gomes, Priscila Karina Siqueira, Rodrigo Roger Vitorino, Eduardo Gomes de Mendonça, Maria Goreti de Almeida Oliveira, Rodrigo Siqueira-Batista

**Affiliations:** 1 Centro Universitário Serra dos Órgãos (UNIFESO), Teresópolis, RJ, Brasil; 2 Faculdade de Ciências Médicas e da Saúde de Juiz de Fora (SUPREMA), Juiz de Fora, MG, Brasil; 3 Universidade Federal de Viçosa (UFV), Viçosa, MG, Brasil; 4 Universidade Federal do Rio de Janeiro (UFRJ), Rio de Janeiro, RJ, Brasil

## Abstract

Erucism is a skin reaction to envenomation from certain poisonous caterpillar bristles. In Brazil, most reports of erucism provoked by *Lonomia* caterpillars are from the southern region. Most manifestations of erucism are local and include burning pain, itching, local hyperthermia and, rarely, blisters (benign symptoms with spontaneous regression in a few hours). General symptoms such as nausea and vomiting, headache, fever, myalgia, abdominal pain and conjunctivitis may also occur. Uncommon symptoms include arthritis, coagulation disorders (manifested as bruising and bleeding), intracerebral hemorrhage and acute renal failure, which comprise serious complications. The present study reports the case of 60-year-old patient from Rio de Janeiro state, Brazil, who came into contact with a caterpillar and developed, a few days later, chronic renal disease.

## Background

Recently lepidopterans of the species *Lonomia obliqua* (order: *Lepidoptera*; family: *Saturniidae*) have been extensively studied due to the severe manifestations provoked by contact with their larval forms (caterpillars), such symptoms are known as erucism. These stinging caterpillars have bristles filled with toxins, which are able to cause lesions, blood and kidney disorders [1,2]. Two species are directly involved and may cause serious or fatal harm to humans, *Lonomia obliqua* and *Lonomia achelous*[2-6].

This article reports the case of patient who came into contact with a caterpillar and developed, a few days later, chronic renal disease.

## Case presentation and discussion

A 60-year-old black man – born in the Rio de Janeiro city and living in Teresópolis, RJ, Brazil – reported that when he was taking the mail from his mailbox, he accidentally put his left arm on six specimens of light green and brown caterpillars, which had about 5 cm length. The animals were crushed by the arm of the man, who, immediately, withdrawn it. He washed the arm using soap and water, applied alcohol to the affected area and also took an anti-allergy drug (could not say which).

The site affected by the caterpillars became swollen and after 20 minutes a burning sensation started. The victim also noticed a painful lymph node in the ipsilateral axilla, which disappeared spontaneously shortly after. In the same night, after dinner, he had abdominal distension associated with discomfort and vomiting. He did not take medicines to relieve the symptoms. As there was improvement, he laid down to sleep. After that, the man woke up at dawn feeling an intense abdominal pain (flank region) and arthralgia (upper and lower limbs), which became worse when he attempted to move.

Subsequently, the patient looked for medical assistance and went to a local hospital. His clinical picture was described as pain associated with sudden anuria. He was admitted to the hospital to treatment. The man reported a previous history of nephrolithiasis and received treatment with saline solution, analgesics and urinary catheter to provide relief. He also received treatment for arterial hypertension with atenolol, 50 mg/day; until this time there was no evidence of kidney injury.

During the hospitalization, the patient had developed diffuse edema, flank pain and the anuria remained, despite of use of intravenous hydration and furosemide infusion. Laboratory and imaging tests were performed (Tables 1 and 2).

**Table 1:** Report of laboratory tests

**Tests**	**Hospitalization days**					**Benchmarks**
	**4**^**th **^**day**	**7**^**th **^**day**	**10**^**th **^**day**	**14**^**th **^**day**	**16**^**th **^**day**	
Leukocytes	18.000	10.800	–	14.800	–	5.000-11.000/mm^3^
Basophils	0	0	–	0	–	0-1%
Eosinophils	1	0	–	0	–	1-5%
Myelocytes	0	0	–	0	–	0%
Metamyelocytes	0	0	–	0	–	0%
Neutrophil/Ban	7	3	–	7	–	1-5%
Neutrophil/Seg	–	74	–	71	–	45-70%
Lymphocytes	14	21	–	19	–	20-45%
Monocytes	5	2	–	3	–	4-10%
Erythrocyte	3.83	2.79	–	2.8	–	3.80-5.20 × 10^4^/mm^3^
Hematocrit	34	24.6	24.4	25.9	27.7	36-50%
MCV	89	88.1	89.9	–	–	80-100 fL
MCH	30	30.3	29.8	–	–	28-32 pg
Platelets	154.000	111.000	148.000	278.000	–	150-400 × 10^3^/mm^3^
PR	–	–	17.6″	15.75″	–	12.7-15.4″
aPTT	–	–	56″	44″	–	26.3-39.4″
Sodium	132	133	135	136	–	135-145 mEq/L
Potassium	7.8	4.8	4.2	4.3	–	3.5-4.5 mEq/L
Urea	190	145	115	113	184	10-50 mg/dL
Creatinine	7.8	9.6	7.8	7.6	11.9	0.6-1.2 mg/dL
ESR	–	46	–	–	–	up to 20 mm/h
CT	–	7′	–	–	–	5-10 min
BT	–	1′	–	–	–	< 7.1 min
c-ANCA	–	Negative	–	–	–	Negative
p-ANCA	–	Negative	–	–	–	Negative
Blood glucose	–	112	–	101	–	70-125 mg/dL

**Source**: patient’s records.

*CT*: coagulation time; *BT*: bleeding time; *ESR*: erythrocyte sedimentation rate; *PR*: prothrombin ratio; *aPTT*: activated partial thromboplastin time; *MCV*: mean corpuscular volume; *MCH*: mean corpuscular hemoglobin; *c-ANCA*: cytoplasmic antineutrophil cytoplasmic antibodies; *p-ANCA*: perinuclear antineutrophil cytoplasmic antibodies.

**Table 2:** Medical imaging tests

**Test**	**Changes**
Kidney and urinary system ultrasonography	Enlarged kidneys
Computed tomography	Inflammatory infiltration of the perinephric fat, uncomplicated renal calculus on the right and a small amount of fluid in the pelvis

**Source:** patient’s records.

After two more days, the patient was transferred to the Hospital das Clínicas de Teresópolis Costantino Ottaviano (HCTCO), where he remained for forty-five days due to hemodialysis treatment. Four days after the admission to HCTCO, he remembered the episode with the caterpillars in the morning before the painful crisis and anuria. That was the moment when the suspicion of erucism by *Lonomia* aroused (seven days after the accident).

The use of SALon was not indicated because the patient did not meet the criteria for it, particularly due to the time elapsed since the accident, hospital care for more than 10 hours and less than or equal to 36 hours [1]. During the admission the patient showed bruises scattered on upper and lower limbs and two voluminous cases of melena. After those events, there was no spontaneous recovery of renal function, which progressed to chronic renal disease (CRD), stage V – end-stage renal disease, when the glomerular filtration rate is below 15% of normal and the patient needs, invariably, renal replacement therapy.

*Lonomia* caterpillar has about six to seven centimeters long, and its color ranges from light brownish-green to yellowish-brown with three longitudinal stripes of dark-brown [4]. Its body is covered with bristles that contain toxins. The transformation to an adult moth occurs after a in ten weeks after three to six months of larval life [7,8].

*Lonomia* is found throughout Brazil, however, numerous registered cases of erucism occurred in the southern region, mainly in Rio Grande do Sul and Santa Catarina states, and were attributed to *L. obliqua*[9,10]. In recent years, there have been accidents in Minas Gerais, Goiás, Maranhão and Rio de Janeiro states [1,4,7].

Erucism caused by *Lonomia* is uncommon in the state of Rio de Janeiro. Therefore, the present study is one of the first cases reported in the state. The increased rate of envenomations – especially in areas where they were not previously described – has been attributed to deforestation of indigenous trees, natural habitat of caterpillars, which are forced migrating to fruit trees in urban areas [1,8].

The symptoms of *Lonomia* envenomation range from local cutaneous manifestations to serious and potentially fatal systemic reactions [11]. General symptoms such as headache, unspecific indisposition, fever, nausea, vomit, arthralgia, myalgia, conjunctivitis and abdominal pain vary depending on the species involved, the intensity of the contact and the victim’s response [7,8,12,13]. Hemorrhagic syndrome and acute renal failure (ARF) are unusual outcomes, but potentially fatal [7,14-18].

The pathophysiological mechanisms of ARF in *Lonomia* envenomig are not clear yet. Probably, there is a relation between renal ischemia and systemic hypotension and/or fibrin deposition in glomerular capillaries [19-21]. Another hypothesis is that venom components may act directly on the kidneys [22,23].

*Lonomia* spp. venom is rich in several toxins that have procoagulant and fibrinolytic activities, which can significantly affect the blood coagulation process. For example, the enzyme lonofibrase is able to trigger a hemorrhagic syndrome similar to disseminated intravascular coagulation (DIC) by increasing fibrinogen degradation products and decreasing plasminogen, fibrinogen and factor XIII [1,5,17,24-26]. *Lonomia obliqua* venom contains several lipocalins (protein group that transports hydrophobic molecules), among which is the *Lonomia obliqua* prothrombin activator protein (Lopap), involved in the increase of expression of adhesion molecules on cellular surface [27-30].

Erucism is diagnosed based on history of contact with the caterpillar and corroborated by data from laboratory tests. Laboratory abnormalities include slightly low platelet count, high urea and creatinine levels, slight increase of total bilirubin, and augmentation of indirect bilirubin, free hemoglobin and haptoglobin decrease in cases of hemolysis [11].

Treatment consists of washing the affected area with cold water, cold compresses, local anesthetic infiltration using lidocaine 2% and topical corticosteroids. In case of bleeding, the patient should be kept resting in order to avoid traumatic intervention [5,11]. The antilonomic serum (SALon) is indicated according to the severity of the accident, and its early administration prevents bleeding manifestations that start from one to ten days after the contact depending on its intensity and location [12,17,31-35].

## Conclusion

The present study comprises an important report concerning the occurrence of *Lonomia* accidents in Rio de Janeiro, Brazil, a very uncommon situation in the area. This case also emphasizes the unusual progression of the envenomation to CRD, which, to the best of our knowledge, was not previously reported in the literature.

## Consent

Informed consent was obtained from the patient for publication of this case report. The research project was submitted for analysis and approved by the UNIFESO Ethical Committee for research with human subjects (CEP), in accordance with *Resolução 196/96* and *Resolução 251*–*97* of the Brazilian National Health Council (*Conselho Nacional de Saúde do Brasil*).

## Abbreviations

CRD: Chronic renal disease; ARF: Acute renal failure; DIC: Disseminated intravascular coagulation; SALon: Antilonomic serum.

## Competing interests

The authors declare that there are no competing interests.

## Authors’ contributions

PAS, TCF, RCS and RCA described the case and drafted the first version of the article. APG, PKS, RRV, EGM, MGAO and RS-B made critical revision of the text. All authors read and approved the final manuscript.

## References

[B1] Correa MS Siqueira-Batista R Gomes AP Franco-Barbosa A Verzola ACA Oliveira FRQ Squeff FA Motta-Leal-Filho JM Tavares RH Amorim DS De-Maria-Moreira NL Santos SS Erucismo por *Lonomia* spp em Teresópolis, RJ, Brasil: relato de um caso provável e revisão da literatura Rev Soc Bras Med Trop 2004 37 5 418 421 15361962 10.1590/s0037-86822004000500011

[B2] Brasil. Ministério da Saúde Manual de diagnóstico e tratamento de acidentes por animais peçonhentos. 2ª edição 2001 Brasília: Fundação Nacional de Saúde

[B3] Kowacs PA Cardoso J Entres M Novak EM Werneck LC Fatal intracerebral hemorrhage secondary to *Lonomia obliqua* caterpillar envenoming: case report Arq Neuropsiquiatr 2006 64 4 1030 1032 17221019 10.1590/s0004-282x2006000600029

[B4] Lisete ML Corseuil E Aspectos morfológicos de *Lonomia obliqua* walker (Lepidoptera: Saturniidae) Neotrop Entomol 2001 30 3 373 378

[B5] Veiga ABG Caracterização molecular dos componentes do veneno de Lonomia obliqua: genes expressos e princípios ativos envolvidos nos distúrbios da coagulação e da fibrinólise Dissertação de doutorado 2005 Universidade Federal do Rio Grande do Sul [http://www.lume.ufrgs.br/bitstream/handle/10183/6566/000531846.pdf?sequence=1]

[B6] Instituto Butantan Acidentes por animais peçonhentos[http://www.butantan.gov.br]

[B7] Gamborgi GP Insuficiência renal aguda em pacientes após acidente com lagarta da espécie – Lonomia obliqua Dissertação de Mestrado 2004 Universidade Federal do Rio Grande do Sul [http://www.lume.ufrgs.br/handle/10183/7807]

[B8] Hossler EW Caterpillars and moths – Part I. Dermatologic manifestations of encounters with Lepidoptera J Am Acad Dermatol 2010 62 1 1 10 20082886 10.1016/j.jaad.2009.08.060

[B9] Garcia CM Danni-Oliveira IM Ocorrência de acidentes provocados por *Lonomia obliqua* Walker, no Estado do Paraná, no período de 1989 a 2001 Rev Soc Bras Med Trop 2007 40 2 242 246 17568899 10.1590/s0037-86822007000200021

[B10] Rubio GBG Vigilância epidemiológica da distribuição da lagarta *Lonomia obliqua* Walker, 1855, no Estado do Paraná: Brasil. Rio de Janeiro Cad Saúde Pública 2001 17 4 1036 11514886 10.1590/s0102-311x2001000400039

[B11] Hossler EW Caterpillars and moths Dermatol Ther 2009 22 4 353 366 19580579 10.1111/j.1529-8019.2009.01247.x

[B12] Centro de Informações Toxicológicas de Santa Catarina Lonomia 2008 [http://www.cit.sc.gov.br/index.php?p=monografias]

[B13] Bohrer CB Reck Junior J Fernandes D Sordi R Guimarães JA Assreuy J Termignoni C Kallikrein-kinin system activation by *Lonomia obliqua* caterpillar bristles: involvement in edema and hypotension responses to envenomation Toxicon 2007 49 5 663 669 17188732 10.1016/j.toxicon.2006.11.005

[B14] Chudzinski-Tavassi AM Carrijo-Carvalho LC Biochemical and biological properties of *Lonomia obliqua* bristle extract J Venom Anim Toxins incl Trop Dis 2006 12 2 156 171

[B15] Prezoto BC Maffei FH Mattar L Chudzinski-Tavassi AM Curi PR Antithrombotic effect of Lonomia obliqua caterpillar bristle extract on experimental venous thrombosis Braz J Med Biol Res 2002 35 6 703 712 12045836 10.1590/s0100-879x2002000600011

[B16] Berger M Reck J Jr Terra RM Pinto AF Termignoni C Guimarães JA *Lonomia obliqua* caterpillar envenomation causes platelet hypoaggregation and blood incoagulability in rats Toxicon 2010 55 1 33 44 19577588 10.1016/j.toxicon.2009.06.033

[B17] Chan K Lee A Onell R Etches W Nahirniak S Bagshaw SM Larratt LM Caterpillar-induced bleeding syndrome in a returning traveler Canadian Med Assoc J 2008 179 2 158 161 10.1503/cmaj.071844 PMC2443214 18625988

[B18] Alvarez Flores MP Zannin M Chudzinski-Tavassi AM New insight into the mechanism of *Lonomia obliqua* envenoming: toxin involvement and molecular approach Pathophysiol Haemost Thromb 2010 37 1 1 16 20714126 10.1159/000320067

[B19] Duarte CA Caovilla J Lorini I Lorini D Mantovani G Sumida J Manfre PC Silveira RC Moura SP Insuficiência renal aguda por acidentes com lagartas J Bras Nefrol 1990 12 4 184 187

[B20] Mendonça RZ Greco KN Sousa APB Moraes RHP Astray RM Pereira CA Enhancing effect of a protein from *Lonomia obliqua* hemolymph on recombinant protein production Cytotechnology 2008 57 1 83 91 19003176 10.1007/s10616-008-9141-4 PMC2553636

[B21] Pinto AF Dragulev B Guimarães JA Fox JW Novel perspectives on the pathogenesis of *Lonomia obliqua* caterpillar envenomation based on assessment of host response by gene expression analysis Toxicon 2008 51 6 1119 1128 18367225 10.1016/j.toxicon.2008.01.010

[B22] Abdulkader RC Barbaro KC Barros EJ Burdmann EA Nephrotoxicity of insect and spider venoms in Latin America Semin Nephrol 2008 28 4 373 382 18620960 10.1016/j.semnephrol.2008.04.006

[B23] Fan HW Cardoso JLC Olmos RD Almeida FJ Viana RP Martinez APP Síndrome hemorrágica e insuficiência renal aguda em uma mulher grávida após contato com lagartas *Lonomia*: relato de caso Rev Inst Med Trop São Paulo 1998 40 2 119 120 9755567 10.1590/s0036-46651998000200010

[B24] Carrijo-Carvalho LC Chudzinski-Tavassi AM The venom of the *Lonomia* caterpillar: an overview Toxicon 2007 49 6 741 757 17320134 10.1016/j.toxicon.2006.11.033

[B25] Berger M Reck J Jr Terra RM da Silva WO B Santi L Pinto AF Vainstein MH Termignoni C Guimarães JÁ *Lonomia obliqua* venomous secretion induces human platelet adhesion and aggregation J Thromb Thrombolysis 2010 30 3 300 310 20157842 10.1007/s11239-010-0449-5

[B26] Siqueira-Batista R Gomes P Calixto-Lima L Vitorino RR Perez MCA Mendonça EG Oliveira MGA Geller M Sepse: atualidades e perspectivas Rev Bras Ter Intensiva 2011 23 2 207 216 25299722

[B27] Lucena S Guerrero B Salazar AM Gil A Arocha-Piñango CL Degradation of extracellular matrix proteins (fibronectin, vitronectin and laminin) by serine-proteinases isolated from *Lonomia achelous* caterpillar hemolymph Blood Coagul Fibrinolysis 2006 17 6 427 435 16905945 10.1097/01.mbc.0000240914.78768.7c

[B28] Reis CV Andrade SA Ramos OH Ramos CR Ho PL Batista IF Chudzinski-Tavassi AM Lopap, a prothrombin activator from *Lonomia obliqua* belonging to the lipocalin family: recombinant production, biochemical characterization and structure-function insights Biochem J 2006 398 Pt2 295 302 16734589 10.1042/BJ20060325 PMC1550302

[B29] Seibert CS Santoro ML Tambourgi DV Sampaio SC Takahashi HK Peres CM Curi R Sano-Martins IS *Lonomia obliqua* (*Lepidoptera*, *Saturniidae*) caterpillar bristle extract induces direct lysis by cleaving erythrocyte membrane glycoproteins Toxicon 2010 55 7 1323 1330 20152852 10.1016/j.toxicon.2010.02.003

[B30] Carrijo-Carvalho LC Maria DA Ventura JS Morais KLP Melo RL Rodrigues CJ Chudzinski-Tavassi AM A lipocalin-derived peptide modulating fibroblasts and extracellular matrix proteins J Toxicol 2012 [http://www.hindawi.com/journals/jt/2012/325250/] 10.1155/2012/325250 PMC3379166 22737165

[B31] Walter GM Acidente com Lonomia: tratamento com soro antilonômico e insuficiência renal aguda Tese de Mestrado 1999 Universidade Federal do Rio Grande do Sul [http://www.lume.ufrgs.br/handle/10183/26141]

[B32] Pinto AF Berger M Reck J Jr Terra RM Guimarães JA Lonomia obliqua venom: In vivo effects and molecular aspects associated with the hemorrhagic syndrome Toxicon 2010 56 7 1103 1112 20114060 10.1016/j.toxicon.2010.01.013

[B33] Arocha-Piñango CL Guerrero B *Lonomia* genus caterpillar envenomation: clinical and biological aspects Haemostasis 2001 31 3–6 288 293 11910197 10.1159/000048075

[B34] Centro de Informação Toxicológica do Rio Grande do Sul Manual de Diagnóstico e Tratamento de Acidentes por Lonomia 1999 [http://www.bvsde.paho.org/bvstox/p/fulltext/lonomia/lonomia.pdf]

[B35] Gonçalves LR Sousa-e-Silva MC Tomy SC Sano-Martins IS Efficacy of serum therapy on the treatment of rats experimentally envenomed by bristle extract of the caterpillar *Lonomia obliqua*: comparison to epsilon-aminocaproic acid therapy Toxicon 2007 50 3 349 356 17537473 10.1016/j.toxicon.2007.04.004

